# Supplementary material for: SCUBE1 promotes pulmonary artery smooth muscle cell proliferation and migration in acute pulmonary embolism by modulating BMP7
Source: PeerJ. 2024 Jan 19;12:e16719. doi: 10.7717/peerj.16719 (PMC10802153; doi:10.7717/peerj.16719)
Supplement: Supplemental Information 2 [file peerj-12-16719-s002.docx]

SCUBE1





GAPDH





SCUBE1





GAPDH





PCNA





GAPDH





BMP7





GAPDH





SCUBE1





BMP7





BMP7





BMP7





SCUBE1





BMP7





GAPDH





BMP7





GAPDH





PCNA





GAPDH
